# Is Insanity a Disease of the Blood?

**Published:** 1848-10-01

**Authors:** 


ART. V.—*Insanity Tested by Science, as shown to be a Disease rarely Connected with Permanent Organic Lesion of the Brain, and on that account far more Susceptible of Cure than has hitherto been Supposed.* By C. M. BURNETT, M.D. 1 vol. 8vo. 1848.

*Observations on the Proximate Cause of Insanity.* By JAMES SHEPPARD, M.R.C.S.L. *Being an Attempt to Prove that Insanity is dependent on a Morbid Condition of the Blood.* 1 vol. 12mo.

MEDICINE, like every other speculative science, must ever be exposed to the difficulties which arise from the unsettled ground upon which it stands. The exact sciences, which are based upon a mathematical foundation, are alone safe from the ever-changing winds of speculative opinion. They stand upon the rock of fixed and certain principles, and every advance from the first datum must be as clearly and strictly proved as the point from which they start. Now, this exactness of investigation belongs only to those sciences which are strictly mathematical. Chemistry, indeed, might put in her claim to rank with the exact sciences, were we further advanced in knowledge of elementary substances; but until then, we must be satisfied to place it the nearest of all others to those which are strictly inductive. An exact science, or one which is based upon principles which are capable of proof, and are universally allowed to be truths, medicine is not, and we never can hope to raise it to this rank. Its first principles are not settled, and almost all the reasoning of its professors, from what they call *principles*, is purely hypothetical.

To this cause we must ascribe the many sects into which it has, from the days of Hippocrates to the present, been divided—the empirical, the rational, the methodical, the galenical, the chemical, and the mechanical, with their almost infinite subdivisions: the humoral pathology—the hippocratic system, the stahlian doctrines, and those of Hoffman; and for the same reason, we must not at any time be surprised if we are brought back to the by-gone theories of our fathers, and some old system, which in the cycle of medical history has almost been forgotten, is revived, to supersede the prevailing opinions. Signs of such changes are not wanting at the present time. Even the stahlian hypothesis, so absurd in many of its imaginings, has been partially revived by the Homœopathists of our day; and the humoral pathology which has been mixed up, more or less, with every system, before and since the time of Boerhaave, but which suffered an almost total overthrow by Cullen's theory of spasm, is beginning to have its advocates, and lift up its head once more among the fashionable systems of medicine. We have before us two well-written treatises on the humoral pathology as applied to explain the phenomena of mental disease, and lead to a more certain and rational practice. One of these was, indeed, written four years ago, and the other appeared this year; but the object of both Mr. Sheppard and Dr. Burnett is the same, which is, to prove that insanity, in all its phases, is to be traced primarily to a diseased condition of the blood. Before entering upon an examination of these works, we consider it our duty to confess ourselves free from theoretic prejudice. A long experience of the practice of medicine has had the effect upon our minds of shaking the sectarian tendencies of our youth, and we have lived to see that truth lies in no particular system, and no sect has a right to claim it as its own peculiar property. Even in the stahlian doctrines, as revived by Hahneman, and especially his disciples, we see much that is valuable. The *vis medicatrix naturæ,* which is the first principle of their systems, is a well-established fact; and there can be no doubt that it exerts itself in the removal of all internal diseases, in the same manner that it does in the expulsion of a thorn from the flesh, by the processes of inflammation and suppuration; and if nature would but confine her kindly efforts within due and safe bounds, we might always regard symptoms as her voice, crying, come and help me. Now, so far, we think, the homœopathists have truth with them; but when they affirm that always we are to encourage and never to control the operation of this principle, we stand ready to join issue, and contend that though the inflammation of a delicate organ may be regarded as a provision for the expulsion of some extraneous matter from the system, *that* inflammation may proceed too far, and endanger the organ it has come to help: to save which, depletion and other antiphlogistic remedies must be resorted to. What we do in the search after truth in our examination of one system, we would do in all—pick and cull what we conceive to be valuable, and reject the rest. With this determination, we can look with complacency upon every novelty, and are not ruffled and made uncomfortable in mind by contact with any of the antagonist pathologies of the day; and feeling at this moment that we possess a double portion of this amiable spirit, we are glad to come in collision with two authors of so much merit as Mr. Sheppard and Dr. Burnett, hoping, in our examination of their works, to do full justice to their opinions, and to extract therefrom much information which will be valuable to ourselves and our readers.

The little work of Mr. Sheppard is intended to promote inquiry, rather than establish a theory. He writes as if it *may be true* that the blood is primarily affected in every disease, and is consequently the seat of all mental affections. He does not dogmatize, but puts forward his facts as presumptive, rather than positive testimony to the soundness of his opinions; and therefore his work may be regarded as a modest inquirer after truth, asking questions, rather than teaching *ex cathedrâ.* But, though the author of the work under consideration comes before us to ask—first, whether the mind is capable of acting on the blood so as to alter its constitution? and, secondly, whether any morbid conditions of the blood can so act on the mind as to interfere with its perfect development? and, thirdly, whether the blood, in certain morbid conditions, can so interfere with the development of mind as to cause insanity? He boldly asserts his belief in the value of that system of medicine which he takes as the ground-work of his investigation, and expresses his conviction, arising from daily observation, "that the humoral pathology is rapidly regaining ground, and that the blood in which is the life, is the structure that is primarily affected in every disease."

Confining himself, however, to the question of insanity as the product of diseased blood, our author commences his attack upon the existing opinions, by showing from the writings of the most celebrated physicians —1st. That in very few instances can we detect the cause of insanity in any alteration of the cerebral tissues. 2nd. That mania *per se*, seldom destroys life. 3rd. That while there is an absence of cerebral lesion, there is almost constantly found more or less evidence of disease in other organs. And lastly, that where a change of structure has been found in the brain, there is reason to ascribe it to the same causes which in other organs produced the same result; all which he endeavours to prove from the writings of Sir H. Halford, Dr. Mason Good, Dr. Sutherland, Georget, Dr. Pritchard, and M. Esquirol, who found that, at the Saltpêtrière, secondary organic lesions of the thoracic and abdominal viscera were the most frequent phenomena discovered after death, and that out of 176 patients, only six exhibited any cerebral disease—and again, that of upwards of 600 examinations after death, three-eighths died of diseases of the abdomen, two-eighths of diseases of the chest, and three-eighths of alterations of the brain and its membranes.

Conceiving that from this evidence he has proved that insanity is often developed without any change of the cerebral structure, or of its membranes, or of the cranium, discoverable after death, our author tells us that we must conclude—1st. That insanity may result either from a disease, or impairment of the entire mind, or of what is termed its powers or faculties. Or 2nd. That unappreciable lesions of structure are equal to the production of insanity. Or 3rd. That morbid conditions of the nervous system *generally* can produce insanity. Or, lastly, that insanity, when developed, results from the action of a morbid condition of the circulating fluid. These four propositions are then examined, and the three first being found untenable, the conclusion of course is, that the last is the only cause of mental disease—that insanity, when developed, results *manifestly* at times from a morbid condition of the blood; and that there is, at least, presumptive evidence that it *may always* depend on a morbid condition of the circulating fluid.

Having arrived at this conclusion, we will leave Mr. Sheppard for a short period, and turn to the more philosophical and elaborate treatise of Dr. Burnett—the object of which is, likewise, to prove that insanity is a disease, not primarily of the brain, or mind, as developed through that organ, but of the blood. We will, however, give his opinion as shortly expressed in his own words:—

"We shall at once proceed to show that both reason and science favour the idea that insanity is not, and ought not in the first instance, and often to the very last, to be regarded as a disease of the brain, but as a disease floating in the blood, having no fixed or local character, but producing the morbid phenomena which are comprehended under the title of insanity; it arises from a derangement or mal-assimilation of those particular materials of the blood, carbon and phosphorus, which constitute the bulk of the elementary tissue of the brain and nervous system generally. When, therefore, we say we believe the disease to be in the blood, we consider it to exist there in the form either of deteriorated, or wrongly constructed chemical compounds. In this sense it must be the *seat*, although Fletcher and Broussais consider it only in the light of the *vehicle* of disease."

This being the case shortly stated, we will now proceed to show the manner in which it is sustained. Here we find that much the same line of argument is taken as that which we noticed in the work of Mr. Sheppard; and therefore, passing by much discursive preliminary matter, we will proceed at once to notice what is affirmed respecting the morbid condition of the brain of deceased lunatics, discovered upon dissection; for we consider this as the groundwork of any investigation of the cause of mental disease. If it be true that insanity is compatible with soundness of brain, and that in the greater number of cases examined no cerebral lesion can be detected, then we should indeed begin to look elsewhere for the seat of the disease; and feeling the importance of establishing this fact as the premise of the argument, we are astonished that in so well-written and elaborate a treatise as that of Dr. Burnett, it is passed by so rapidly, and with no attempt to give more than the *ipse dixit* of a few leading writers upon the subject.

The evidence on the point at issue is scattered throughout the work; but we will place it together, that what is said may have the greatest weight.

At page 12, we find the first allusion to the subject of morbid appearances—following a marginal reference to the subject, which states that numbers of the insane die without any marks of disease in the brain.

This broad assertion is then supported by the evidence of some illustrious names, as Esquirol, Pinel, Georget, Bichat, Haslam, Greding, and Percival; and it is to be regretted that only the results of their practice, or their mere opinions, are given, so that we are not able to test the accuracy or estimate properly the value of this testimony.

But let our author speak for himself:—

"It has been asserted by Esquirol, that nearly one half of the bodies of the insane, that is, three-eighths, die of some affection of the viscera without any corresponding changes in the brain itself, at least which are perceptible as they are in other organs to the eye of sense. Pinel, his great instructor, whose anatomical pursuits were most extensive, held similar opinions. So also did Georget, Bichat, Haslam, Greding, Percival, and many others, from a most extended field of observation, arrive at the same conclusion. In a list of the morbid appearances of 259 dissections of lunatics quoted by Burrows from the work of Scipion Pinel, in which he embraced the labours of Esquirol, Villermai Beauvais, and Schwilgaé, we find 135 presented diseases in the thoracic and abdominal cavities, and 56 showed no morbid appearances whatever. This table gives, therefore, about one-fourth of the whole to diseases of the brain generally, while in one-fifth there was no structural disease anywhere.

"But what is still more remarkable, is the known fact, though we are not aware of its having been pointedly alluded to, that in the whole catalogue of nervous diseases, in the course of many of which the functions of the mind become involved, there is an absence of all morbid appearances in the brain after death—hysteria, catalepsy, chorea, hypochondriasis, epilepsy, idiocy, and similar affections terminating in insanity, and even mania and apoplexy itself are constantly occurring in individuals whose brains after death present no semblance of disease. We ask, could this be possible if the phenomena which so often end in insanity, and which even indicate what we call its worst forms, were the result of structural disease in the brain? We think it could not. We suspect there is as much argument even when disease is actually present, as in cases of ramollissement of the brain, to show that that condition of the organ is not the result of inflammatory action as that it is. What few morbid appearances of the brain which are found to accompany insanity are nine out of ten to be referred to the venous system in that organ, thereby showing that even the mechanical detention of venous blood in the brain—a cause that may be acting for a long period of time upon the structure of the brain, so far as to derange its function, is not attended with such alterations of structure as we see so quickly follow inflammatory diseases. Between insanity and morbid alterations of the brain, says Dr. M'Cormac, there seems no necessary connexion. It is common to discover them after death, without any preceding mental disturbance, as well as to witness the brains of the insane without any appreciable lesion. There must surely, therefore, be some necessity, if we hope to be successful in the cure of this awful malady, that we trace the evil to a cause which may be acting slowly but surely in the accomplishment of the melancholy result, long before they can be directly pointed to in the brain as actually existing in that organ."

Again, at page 56, the author says,—

"We believe that the brain, as a vital organ, is far less frequently than any other involved in structural disease of any kind, and in those diseases of the adynamic form, so typical of insanity, still less frequently. This we shall prove by several facts. But first, it may be inferred from the known power the brain possesses of repairing injuries done to it, and of resisting the invasion of organic disease which such injuries would be likely to predispose it to. There are numerous instances where injury and compression of this organ has been neither attended nor followed by any morbid symptoms whatever. The memorable case mentioned by Sir Astley Cooper, which was operated on by Mr. Cline, shows that even morbid symptoms may follow a mechanical injury, and, after continuing above a year, may be removed without any detriment to the mental or bodily functions. A sailor fell from a mast, and received a blow on his head, which produced a depression of the skull and symptoms which deprived him of all mental, and almost all physical power. He took food and he breathed, but little more than this did he do for thirteen months, when Mr. Cline trephined the skull in that part where the depression had occurred, and having removed the pressure, he almost immediately spoke, and only a week afterwards his mind had almost completely returned, when he could converse and move about nearly as well as he did before the accident occurred. He remembered now all the circumstances of his being pressed and carried down to Plymouth; but from that period till the operation was performed his mind remained a perfect blank; soon after which, it was restored to healthy action. Surely, if the brain is easily destroyed by injury, the man ought never to have recovered his senses, even if he did his life.

"But, secondly, as regards the power of resisting the invasion of organic disease, the brain is far more able to bear the action of causes which produce inflammation in other organs, without becoming itself so affected. If this observation applies to inflammatory diseases, it is still more forcibly applicable to what are called nervous and mental diseases, which are æsthenic in character, and in which few, if any, of the true symptoms of inflammatory diseases are present. Pathologists have examined the brains of lunatics, expecting to find the ordinary sequels which in those organs are found to characterize inflammatory action; yet they find no such appearances. They do not go on to examine the substance of the brain by chemical analysis; if they did, they would find the deficiency to rest here. It has been clearly proved that the nervous matter of the brains of the insane is deficient in phosphorus; and, in the deficiency of this essential ingredient in the normal constitution of cerebral matter, no surprise ought to take place that this substance is incapable of executing the office of the mind. How far this degenerate condition of the cerebral mass depends upon, or is the cause of, that particular state of the venous circulation in the brain which we call congestion, and which so often attends those diseases which are comprehended under the general term insanity, is a very interesting question. In some forms of mental disease, after death, it is true, we occasionally meet with an alteration in the consistence of the brain, which is comprehended in the term 'ramollissement,' and it is extremely doubtful whether this peculiar appearance is really the result of inflammatory action in the brain. All the symptoms during life most decidedly negative the idea. M. Rostan thought this disease was not inflammatory, but one *sui generis*. It is certainly a very different disease from that softening which is the result of true inflammation of the brain, where no symptoms of insanity are manifested. We contend that it is nothing more than the result of an atonic state of the secernent vessels, by which the cerebral globules become chemically deficient, and are consequently soft. In other words, it is not disorganization, but malassimilation."

At page 59, the following passage occurs:—

"In many old cases of insanity, there can be no doubt that those which are looked upon as incurable cases, on account of some preconceived notion that, from the long duration of the malady, there is organic mischief in the brain, have really no more structural disease there than they had the first day of the manifestation of any morbid symptoms. Our own experience most incontrovertibly assures us of this fact. We have examined the heads of those who have died insane, after having manifested the morbid symptoms for many years, and found none of the appearances referred to inflammatory action, or even any appearance whatever of a morbid character.

"A gentleman was placed under my care who had been for some months under the care of one of the most celebrated physicians for the insane in Paris. It was the opinion of this physician that there was ramollissement of the substance of the brain. He had loss of memory and speech to a great extent, and there was a paralytic contraction of the flexor muscles of the legs. This occurred six years ago, yet he is still living, and has lost all the contraction of the limbs, has great additional muscular strength, and can talk better, and even sings frequently. Could this be so if the brain had undergone six years ago a softening of its structure from inflammation? This case shows either that he was not the subject of ramollissement, or, if he was, that that ramollissement which is sometimes associated with insanity is not an inflammatory disease, but one of malassimilation of the cerebral globules, depending upon very different causes.

"To mention another case: a poor man was married at the age of nineteen, soon after which his wife became unfaithful to him, and he went out of his mind, in which state he continued for nearly thirty years in an asylum. His mother, thinking she might undertake the management of the case without danger, took him home to her house, where he resided for a year or two, but from causes possibly connected with a less moderate or appropriate diet, in one of his fits of depression he cut his throat. He was of a sanguineous temperament, and the loss of blood was quite as great as was compatible with life. After much care and anxiety, his life was saved, but the most astonishing fact is yet to be stated; he recovered his right mind, and has remained well, *cum sana mente in corpore sano*, ever since, and this is fifteen years ago. It would be curious to know, after this man had been nearly thirty years in an asylum, what was the opinion of the visiting physician upon a case of so long standing. Could it be possible that the brain, exposed as it was for so many years to the operation of some morbid cause, no doubt latent in the blood, which led to a derangement of mind, could only have been affected functionally—that is to say, in this case curably? And yet it is inconsistent with reason to suppose that the organic structure of the brain was involved; for, if so, how could the man, after thirty years, be restored to all his faculties? How much more probable is it that the brain in this case was only functionally disturbed from a disease existing in the blood; which disease, during the long series of *thirty years*, failed to alter the structure of the brain; and, consequently, when the violent remedy came to be applied, which abstracted so large a quantity of that material in which the disease resided, the mental derangement vanished.

"The success which attends the efforts of many enlightened physicians to restore in some degree the mental power of the idiotic and imbecile is again a verification of the same principle we are contending for. If these poor creatures had organic disease or malformation of the brain, they would manifest no improvement when exposed to the action of those second causes which have been so long denied them; but if the natural organization of the brain has only been arrested, there is both reason and hope that human efforts may partially, though not entirely, restore them.

"This is precisely what has taken place. The efforts of M. Seguin, MM. Fabret and Voisin are being responded to in this and other countries, and we shall soon have the happiness of contemplating the restoration of thousands of our fellow-creatures who are sunk into the most wretched and helpless of all human conditions. It will also be found, that not only can the imbecile and idiotic child be made susceptible of enjoyment, and capable of assisting itself, but even those whose bodies have been preserved to adult age are capable of undergoing considerable physical improvement, and of manifesting greater intelligence. This was remarkably exemplified in the case of a lady, brought to us three years ago, who was thirty-three years of age, and from the period of teething, when she suffered from some convulsive attacks, she never made any mental progress. She could not speak or assist herself in any way, but was fierce in her manner, voracious in her appetite, and destructive of everything placed near her. Her body was emaciated, and she uttered a noise of distress. Her kind and faithful sisters had endeavoured all this time to extend to her the protection of their house; but now their health was giving way, and they were obliged to give it up. Much of this long period had been passed in bed, and often from necessity the windows were closed, and the light was excluded from her room. We soon got up her flesh, and successively she has learnt to walk, to feed herself, to amuse herself with pictures, to dress herself, and the like. She seldom makes any kind of noise, and she evidently knows what she ought or ought not to do. She enjoys the sun and the fire, and has learned to discriminate what is good with considerable ability."

In these extracts, we have endeavoured faithfully to give Dr. Burnett's reasons for supposing that the brain is not the organ implicated in mental diseases—and, arriving at this conclusion, he proceeds to search for the cause elsewhere; which, after a very physiologically correct and careful examination of the blood in its formation, its composition, its uses, and its diseased phenomena, he considers he has discovered in that fluid.

After showing that there is no connexion between insanity and morbid alterations of the brain, our author proceeds to say—

"There is then much experience, and no slight argument, to induce us to direct our inquiry to the condition of the blood in mental diseases. But from close observation, we are convinced that the disease called insanity, though unavoidably connected in some instances with organic lesion, and even destruction of the brain, as after many mechanical injuries, is in four cases out of five, in the first instance, a functional disease, quite unconnected with any morbid alteration or change of structure in the brain; and in many of those four cases it continues through a long series of years, still a functional disease, kept up by mal-assimilation. It is, in fact, according to strict pathology, a disease of the blood, but pre-eminently so, from its non-inflammatory character, preventing the morbid alteration of structure, more or less quickly consequent on inflammatory diseases. We believe that insanity in such cases is immediately caused by the deterioration of the fatty matter of the blood, by which the carbon and the phosphorus are unable to combine in healthy proportions, which substances, in a normal state, it is known form the elementary tissue of the brain and nerves, and which chief constituents fail to make that part of the organism of the body amenable to the operation of the vital and mental principles conveyed in the blood.

"Whether this may arise from causes immediately connected with the processes of primary and secondary assimilation, or whether it is consequent upon a particular state of the venous circulation in the head, is uncertain; but the fact made known by Braconot and Chevreul, that the fatty matter united with phosphorus, which constitutes the essential substance of the brain and nerves, has been found by them in the blood, thus combined, favours the idea that the original fault is in the process of secondary assimilation, by which the carbon and the phosphorus unite with the other matters to form new and abnormal compounds. We, however, incline more to the belief that the true separation of cerebral and nervous matter, however essentially dependent upon healthy secondary assimilation, is, nevertheless, only finally completed in the blood vessels after they have entered those tissues.

"We do not consider that this change can be pathologically or philosophically regarded as organic, because we believe the imperfect assimilated fat globules, after they have entered into the composition of the cerebral tissues, are capable of being removed after their deposition, and replaced by those more normally produced, which change is probably attended with an improved alteration in the manifestation of mind through its organ, the brain.

"The process of deposition and absorption is certainly a natural, as it is a healthy process carried on in the brain in the same way as it is in all other organisms, such as the bones, the muscles, the parenchyma of viscera, &c., and it appears to be a law of the animal economy, which acts independently of the material acted upon, though probably not in the same ratio upon all materials. If, therefore, the materials carried to the part or organ in which they are about to be deposited, are not assimilated in those exact relative proportions which constitute health, this may influence the power which absorption may exercise over them; yet if they produce in the organ to which they are conveyed no permanent alteration of structure, no visible displacement of parts, no change of consistence, weight, or colour, we consider the mal-assimilation to be capable of effecting only a functional derangement in that organ to which such mal-assimilated globules are conveyed.

"The diseases which follow the abstraction, or the redundance, or the morbid combinations of many, if not all the constituent elements of the blood, produce alike functional derangement in all the organic tissues, which, in many instances, are effectually removed by the process of absorption, and a healthy state of things is re-established. Still the functional disease will not remain such in all cases for an unlimited time, if the causes producing that functional disease are still kept up and allowed to operate. There is a period, I believe, when, from the long-continued supply of morbid or mal-assimilated particles, the structure of particular parts becomes changed, and then we have organic disease set up, which can no longer be susceptible of removal. The progressive way in which this change from functional to structural disease is produced, may be accelerated or retarded by those causes which originally produced the morbid action, being continued or discontinued either partially or entirely. It will be our object to show that the diseases essentially called nervous, that is, diseases implicating the healthy function of the brain and nerves, are almost universally and in a far higher degree than other organizations, from first to last, diseases resulting from mal-assimilation of the blood constituents; that the imperfect preparation of alimentary matter, by the stomach and other primary assimilating processes, renders them unable when brought into the living current to take up that position, and to form those metamorphous changes peculiar to vital chemistry, and which, being imperfectly fulfilled, lead to the building up of parenchymatous structures, which are chemically deficient, and consequently unable to perform with health and vigour the functions naturally assigned to them. A better example to illustrate this cannot be given than that which takes place in that particular condition of the nervous system which is termed Cretinism. Here we have a deficient state of the brain, as regards its chemical component parts; the phosphorus has been shown to be deficient to a great extent, and imperfectly supplied, and the result has been, not a disorganization of the brain, but a morbid display of mental phenomena.

"The skull of the cretin, described by Dr. Reeve, from one he saw in the Museum at Vienna, was not mal-formed; it was simply incomplete. The subject was thirty years of age, yet the fontanelle was not closed. The second set of teeth were not out of their sockets, and none of the bones, nor their processes, were completely formed. The practical conclusions which Dr. Guggenbuhl has lately arrived at are, that the disease of cretinism is not an organic defect of the material organ of the mind, but, in four cases out of five, the disease consists in a due want of bodily vigour, which renders the senses incapable of conveying external impressions to the mind, and not in the non-existence of the mental faculties.

"No cases show more unequivocally than these do, that they result from the application of deteriorated air and food—two of the most important vital stimulants, and that the removal of such cases from within the influence of such injurious causes is succeeded by a train of improved mental phenomena, more or less independent of the age of the individual, which entirely put to silence any hypothesis that assumes that the organization of the brain was mal-formed in the common sense of the word."

This quotation from "the Preliminary Observations" of our author, we think will sufficiently convey his idea. That he is a humoral pathologist there can be no doubt; and if there is any, after perusing the above extracts, we will dispel it by the doctor's own confession:—

"All due allowance," he observes, "being made for the errors inseparable from the dark period of chemical, and indeed all other science, which characterized the first propagation of the humoral pathology, it is nevertheless very generally admitted in the present day, that its tenets were based upon much sounder principles than those of the school that succeeded. And had the scientific mind been as steadily attracted to the subject of late years as it was at first, the high probability is, that much more real progress would have been made in physiology and pathology, than we can now boast of, with all the accumulated advantages which we derive from improvements and discoveries in science, even in this wonderful age."

Now, we must here, also, confess ourselves to be unopposed to a modified humoral pathology. The blood is undoubtedly a great agent in the production of disease. Long before the slightest organic lesion is produced in the solids, in most cases of disease, the blood is deteriorated.

This deterioration may begin in the digestive apparatus, not from any fault in it, but from overloading the stomach with food, or giving it work which is beyond its power to execute properly. The aliment in this case remains undigested, and being undigested, ferments, and entering the blood in this corrupted state, must produce great changes in that fluid. Will any one, then, deny that blood thus deteriorated may be a fertile cause of disease?

But there are other sources of diseased blood besides the digestive apparatus.

The human body is a great laboratory, in which chemical changes are continually going on. The blood, after circulating through the system, is filled with carbonaceous matter, and is the vehicle for all the impurities poured into it by the absorbents. To rid itself of these extraneous substances, the body is provided with ample powers. The skin, the kidneys, the liver, the lungs, are all at work, constantly carrying off the impure and morbific matters contained in the blood; some by chemical changes, as the formation of carbonic acid in the lungs, and others by a power, which the different organs possess, of separating these ingredients from the mass of the circulating fluid, and then casting them out of the system; and so long as this proceeds regularly, and without interruption, *cœteris paribus*, the blood which circulates in the arteries continues pure, and the source of mental and physical vigour; but any interruption to these processes—any obstruction, for instance, in the portal or pulmonaic systems—will fill the blood to excess with carbon, and lead to such an accumulation of impurities, as will produce the most alarming symptoms; or, if continued, destroy life.

That diseased or impure blood may act as a poison upon the nervous system, and produce immediate fatal results, we must acknowledge; but in the generality of diseases it is only indirectly mischievous; and it is at last by some organic changes in the solids that disease is permanently established.

It is in this manner that insanity, in our opinion, is produced. It may be an indirect consequence of diseased blood. There may be, as a remote cause, indigestion, and consequently mal-assimilation; but we believe that, in every instance, the *proximate cause* will be found in some disturbance of the cerebral substance.

And here we deny that Dr. Burnett, or Mr. Sheppard, have proved their case against the morbid anatomists, who assert, that in the generality of cases submitted to the dissecting knife, some lesion can be detected in the brain. We think, if these gentlemen had examined some of the more modern reports of our large asylums, they would have discovered a greater proportion of disturbed brains in the insane, than three-eighths. We would refer them to Dr. Webster's reports of Mr. Lawrance's dissections carried on in Bethlehem Hospital, as published in the Transactions of the Royal Medical and Chirurgical Society of London, where it is stated that, out of 108 autopsies, there were 92 cases of infiltration of the pia-mater; turgidity of the vessels existed in 89; fluid was effused in the ventricles in 67; effusion had taken place in the base of the brain in 39; there was thickening and opacity of the arachnoid coat in 32; bloody points were observed on the cut surfaces of the medullary substances in 27; the colour of the brain appeared changed in 19; and in 17 cases blood was effused within the cranium.

That there are many cases of insanity, in which no trace of cerebral lesion can be detected after death, and that in most of what are called nervous affections there is no perceptible change in the brain or its appendages, we do not deny; but it does not follow, as a proved fact, that no alteration of structure has taken place because its existence cannot be discovered. It must be remembered that the examination of the brain is surrounded by greater difficulties than that of any other organ; the delicacy of its structure and the rapidity of its decomposition present an almost insurmountable barrier to the progress of our knowledge of its anatomical character. We who are of old standing in the profession, can all remember the careless and rapid way that a brain was wont to be dissected and dismissed, and we do not wonder, therefore, that so little was done in former years to throw light upon mental disease by such cursory and defective *post mortem* examinations. We do not dispute the facts recorded by Esquirol and others, but we doubt very much the ability of anatomists in their day to subject the brain to that close and careful scrutiny which it should pass through before we can positively affirm that it has undergone no physical change. Nothing was known, at the period to which we refer, of the fibrous nature of the brain, and most absurd was the manner adopted by all anatomists then in demonstrating that organ. We of the present day have improved our processes and our knowledge, and yet how little, must we all confess, do we know even now of the minute anatomy of that most complicated and interesting part of the human machine.

Seeing, then, the manifold difficulties which stand in the way of the morbid anatomist, as he prosecutes his investigations into the condition of this organ, we are not surprised that in the reports of the eminent persons referred to, there are found instances of mental disease without any appearances, after death, to show that the brain was the peccant part; and we are not at all shaken, by facts such as these, in our opinion that insanity is a disease of the solid medullary substance, or investing membranes of the brain. Knowing that injuries to the brain, even of the slightest kind, will produce insanity; that a little blood thrown out upon its surface, and pressing upon its delicate substance, the minutest change in the coats which envelope it, the most imperceptible alteration in its consistence, too much blood thrown for a limited period into its vessels, and mechanical obstructions of any kind to the free and uninterrupted circulation through its vessels, will overturn the fairest intellect, or disturb the action of the most vigorous mind; and knowing that in a great proportion of the cases examined after death, organic changes are discovered, we cannot but refer the disease in *all* cases to some lesion of the cerebral substance; and when no such changes can be detected, we are more disposed to cast the blame upon our bungling mode of dissecting, or our ignorance of the minute structure of the brain, than doubt for a moment that, as a general rule, insanity is caused by some disturbance of the medullary matter of that organ.

It is supposed, by those who advocate a humoral pathology, that as disease is *primarily* in the blood, any disturbance which may be detected in the solids, after death, is caused *secondarily*, by that abnormal state of the vital fluid—that insanity, for instance, is owing to a deteriorated and corrupt condition of the blood, even before there is any organic lesion, and that, owing to the same cause, we find, at last, that the solids in different parts of the body, equally with the brain, become diseased.

Now, that this is so, we aver, is by no means proved; and from the facts given in the works under review, it is no more evident that insanity and disease of brain are the products of corrupt blood, than that a deteriorated condition of the blood is the effect of a disturbed brain and deranged mind.

Weighing the evidence produced by the partisans of both these hypotheses, we confess that, in our opinion, the latter preponderates over the former. We see persons suffering for years under the influence of indigestion of the worst kind, with blood that must be deteriorated by this cause, circulating through the brain, without any mental disturbance following as a consequence. We have instances innumerable of patients labouring under scorbutic, cancerous, scrofulous, and syphilitic diseases, in all of which the blood is confessedly corrupt; and yet this highly deteriorated fluid goes on circulating for a life-time without producing insanity; and even in many cases of scrofulous disease, is co-existent with a high order of intellect, and a clear and vigorous understanding.

A remarkable fact presents itself to our recollection as we write on this subject. One of the most remarkable features in that most inscrutable of all diseases, the "Asiatic cholera," is the condition of the blood, which, from the moment the collapsed state supervenes, becomes thick, dark, and tar-like in its consistence, and yet it is a very rare thing to see any mental disturbance in that disease, and the intellect, to the last, remains unclouded, even by delirium. Thus we see how much disturbance and deterioration there may be of the blood without any corresponding disturbance of the mind; it may be corrupted to such an extent as to produce the most disastrous consequences, and even endanger life, without in the slightest degree deranging the intellectual powers.

But, if we reverse the experiments, and first of all disturb the brain, and derange its functions, we very soon throw into confusion the whole apparatus designed for the formation and circulation of the blood, and, consequently, must sooner or later affect the composition of that fluid.

We all know how direct and powerful is the sympathetic connexion subsisting between the brain and the stomach; and the mind and the heart, even to the capillary extremities of the arteries. A blow upon the head will in a moment produce vomiting; strong mental emotions will immediately disturb the digestive functions; a disgusting object, or distressing intelligence, or a sudden catastrophe overtaking a hungry man, will directly affect the appetite of the most voracious epicure, and slight mental emotions, as fear, shame, joy, or anger, will instantaneously disturb the whole sanguineous circulation from the heart to the extreme vessels. Now if such transitory impressions upon the brain or the mind are capable of producing such powerful and immediate effects upon the very sources of the blood, we may easily conceive what will be the probable result of long continued irritation, moral or physical. Let any of these causes be kept for a long period in operation, and the result will be, primarily, disease of the heart or stomach, with impaired digestion, mal-assimilation, or irregular circulation; and secondly, corrupted blood: for as good blood is the product of a healthy stomach, bad and deteriorated blood is the certain result of any long continued disturbance of its functions; and as it is shown, that such an abnormal condition of that organ is often a consequence of a disturbance of the brain or the mind, it follows, that insanity may indirectly impair the condition of the circulating fluid, and through it, the whole animal economy. This we find is the consequence of chronic mania, in almost every instance; and if, upon examination after death, we discover lesions in any other organs, as well as in the brain, we ascribe those changes directly to the deteriorated state of the blood, but indirectly to the disturbance which has been going on in the mind and nervous system.

Now, as we before said, a modified humoral pathology is not a system that we oppose. We would not disregard the blood in its several conditions, in treating disease, because humoral pathologists have, in their enthusiastic admiration of their hypothesis, treated the solids with too much contempt and indifference. We believe that blood deteriorated by any cause is a fertile source of disease, and any practitioner of medicine who disregards its abnormal condition, would do so to the manifest injury of his patients. Happily, whatever may be our various opinions upon this subject, we all, in our treatment of disease, do, through the digestive function, as much for the re-establishment of a healthy condition of the vital fluid, as the most ardent humoral pathologist could desire; and upon looking over the treatment of insanity proposed by Dr. Burnett, we find nothing new or strange in his hygienic or therapeutic views. To all that he says of the value of good food and plenty, fresh air, cleanliness and exercise, we heartily reply, Amen; and we believe the various therapeutic agents recommended by him have all been well tested, and many of them admitted among our most valuable means of cure, by most psychological physicians. So far, then, we can row heartily in the same boat together, but when the doctor comes to speak of the moral and restraint system of treating the insane, we find we must scramble out, and take another course.

That moral treatment is of the utmost value, we have every reason, from long continued experience of its benefit in every form almost, of mental disease, to acknowledge with gratitude. It might be supposed by persons little acquainted with the insane, that as the mind is gone, and all that elevates man above the brute is depressed, there can be nothing to work upon in our prosecution of a strictly moral treatment: and, therefore, it can be of no more utility in the management of a lunatic, than it would be in that of a dumb animal. Were this the deplorable condition of the insane, we could quite understand the objection here raised; but it is a most gratifying truth, which our own experience enables us to confirm, that in very few instances, comparatively, is there such a blank in the moral visage, as to defy the application of this principle. There is, in almost every case, some lurking old feeling, or instinct, or inclination, which, in skilful hands, may be used as a fulcrum upon which our lever may rest.

Rarely, we are disposed to hope, even in the worst forms of idiocy, is the mind so completely destroyed as to render nugatory all kindly efforts to restore its powers by moral means; but in all other forms of mental disease there are always, more or less, some remains of moral sense, or intellectual power; and in many cases of monomania, the faculties of perception, memory, and judgment, are not only present, but in the highest vigour, when exercised upon all other matters than that which constitutes the mind's weak point.

Now, to this extent, we agree with Dr. Burnett, but when he grounds upon this fact an argument for the punishment of all criminal lunatics as of responsible agents, we take the liberty, in common with all philanthropists, and jurists, to record our repugnance at the mention of a thing so contrary to the spirit of our religion and laws.

That a lunatic may have great powers of understanding and reasoning upon every subject but that upon which he is mad, we have acknowledged, but surely this cannot make him responsible for the acts committed by him when under the influence of those impressions which constitute his madness—he might, for example, imagine that he was commissioned by God to destroy a certain individual, and as long as the object of his enmity was kept out of view, he might appear as rational and composed, and even as religiously disposed, as other men; but if he were to come in contact with that individual, and in a moment of returning frenzy, influenced by his mordid imagination, destroy him, surely no one would, on the ground of his powers of reasoning on other matters, consider him responsible for this act. However sensible otherwise he might be, here he was mad; and acting under the power of a necessity which he could not avoid—an impulse which he could not control—he feels himself compelled to the commission of the offence, as a wild beast which generally is quiet and manageable, may, in any moment, be impelled by some sudden impulse to attack his keeper.

In such a case as the one mentioned, the individual is an irresponsible being. He knows not that he is doing wrong; indeed, he imagines all the while, that he is doing God service; and, therefore, to treat such an offender as a rational being, and punish him for his act, would be as irrational and wicked as to punish, as a responsible agent, a dog or a horse for killing a man.

But before we allow a criminal to escape justice on the ground of insanity, we ought clearly to ascertain that the act he has committed was a consequence of some erroneous conceit connected with his hallucination. There should be no mistake on this point. A well established connexion should be made out between his acts and his false imaginings, otherwise madmen may always escape the consequence of actions for which they *are responsible* and which they know to be wrong. The lunatic who imagines another man to be in pursuit of his life, and, to protect himself, kills that man, is not for that act responsible; but if this lunatic, in a fit of passion, provoked by the ill treatment of another, should kill that person, then, for that act *he is responsible,* and should not be screened from the consequences by any plea of insanity upon other points. We wish that we had left ourselves space enough to enlarge upon this point, as we deem the question raised by the observations of Dr. Burnett one of the most grave importance. There is nothing that more requires settling than this dispute about the responsibility or irresponsibility of criminal lunatics, as we are in danger, in consequence of the present state of the law and the loose opinions of medical men on the subject, either of protecting every villain on the ground of moral insanity, or subjecting those who know not right from wrong to the punishments of responsible and rational beings. We do not agree with Dr. Burnett, that every mad act should be punished, but we have no wish to screen madmen from the consequences of crimes which they know are wicked and contrary to law.

As Dr. Burnett would hang madmen for murder, as a great moral means of checking such tendencies in others, we are not surprised at his admiration of mechanical restraint as a powerful agent in controlling and curing the insane.

"Nothing," he observes, "can exceed in absurdity the preposterous attempts that have been made to impose on the public mind the idea that the insane are treated without restraint. Why, the very building they are confined in belies the word non-restraint. Is it possible that the public really believe such a thing to be true? and, still more, do they believe, even supposing it to be true, that it is a humane principle? Two causes seem to have conduced mainly to this popular delusion—viz., a desire to remove the evils which the old system of restraint had given rise to, and which had very properly produced the greatest disgust and even horror in the public mind; and a desire to substitute some plan that would attract notice by its antithesis to the one that had preceded. Without any practical knowledge of the success of such a plan, supposing it to be susceptible of application, according to the literal meaning of the word, the simple reflection that it comprehended a plan as ultra in its way as the old extreme system of restraint, should put the public upon their guard before they seek to adopt it so universally, or believe that it can be said truthfully to be practical. We wish we could say that no evil had already arisen from what is called the non-restraint system. We believe it to be full of evil, as must every plan be that assumes to adopt an extreme method. Every branch of the science of medicine testifies that, when we hope for success, we must avoid extremes; and this rule has its advantages, we are fully persuaded, in the treatment of the insane. All see the evils plainly enough that were allowed to continue under what has been called the old system, where those agents, which must be admitted even by the most fastidious non-restraint advocate, have their use. Is it at once to be inferred that because brandy is calculated to produce intoxication, thereby depriving man of his senses, it is incapable of restoring the senses under different circumstances, as, for example, from a state of syncope. We hear of cases where life has been forfeited to the injudicious application of hydropathy. Should we do wisely, if, on this account, we ceased to avail ourselves of water as a useful hygienic agent? What should we argue of that so called philosopher, who, having discovered for the first time that fire, for example, had the property of destroying bodies, proceeded to denounce it, not only as a dangerous, but a useless agent. It may with great certainty be affirmed that the human mind has been permitted to make no discovery, whether relating to the laws or the combination of matter, that has not some use for which it has been made known. It is true we may be living in an age when an agent may be only known to us by its abusive application; but who can say its use has never been applied with advantage? It is, therefore, a gratuitous and disingenuous assertion to say there is no use in restraint; and those who use such expressions lay themselves open to one or other of these charges—either that they are ignorant of the nature of those remedies which they denounce, and which they are all the while hourly but unconsciously using, or else they are really practising a fraud upon their own consciences. This state of things cannot be permanent, and sooner or later the deception will yield to the forcible impulse of truth."

Now we cannot but regard this attack upon those physicians who profess to pursue the non-restraint system, as unjust in the extreme. Our author attempts to place them upon the horns of a dilemma, and compel them to acknowledge that they are either ignorant of the nature of the restraint they are using, or practising a fraud upon themselves; and we may add, though the Doctor does not, as a corollary, a fraud upon the public!

Now we feel bound, as honest defenders of truth, to come at once to the rescue of these unfortunate gentlemen, and, if possible, even at the risk of concentrating the irate Doctor's wrath upon ourselves, to snatch them from the horns on which they are impaled.

In the first place, we do not believe that any advocate of what is, for want of a better term, called "*non-restraint*," ever advanced the wild, visionary, and insane opinion, that all kinds of restraint (using this term according to Dr. Burnett's unfair acceptation of it) upon the perverted wills, and ungovernable passions, and dangerous propensities of the insane, might be, and ought to be indiscriminately dispensed with. Such a preposterous notion never entered the head of any man out of Bedlam, and to prove it, by referring to lunatic asylums, barred windows, athletic attendants, seems to be not only unphilosophical, but, as we regret to say, superfluous twaddle.

The term "non-restraint" was adopted simply to designate the system of treatment, opposed to that which allows the use of coercive mechanical measures—we do not say to the extent which was formerly, to the disgrace of humanity, permitted, but still considerably beyond what, in their opinion, is necessary for the safe keeping and cure of the insane.

These physicians use the term non-restraint as an enlightened Englishman does the words liberty and freedom of the subject, so constantly in his mouth; and, as Mr. Bull never contemplates the thought, while uttering these words, of an *entire and complete liberty*, or freedom from the laws and usages of the country or society, so neither does the most ultra of all non-restraint advocates, entertain for a moment the idea that restraint is to be entirely abandoned; and we might as well charge the Englishman with ignorance of the nature of that liberty he boasts of, or say that, while a slave to laws and opinions, he is deceiving himself with the notion of freedom, as to assert of non-restraint physicians that they are, by the use of the term in question, either ignorant or fraudulent. The public, when it is told of the non-restraint system, fully understands its meaning, and therefore, there is no deception practised upon it; and those physicians who advocate and practise it, do so with the understanding that they have full liberty to adopt all precautionary measures which are necessary for the comfort or the protection of their patients.

From a long experience of the difficulties which hedge up the way of the humane psychological physician, we have arrived at the conclusion that no rule can be laid down, which shall bind us to either of these systems; and that the head of an asylum best fulfils the trust reposed in him by the public, when he leaves himself at perfect liberty to exercise an unbiassed judgment in any difficulty that may present itself.

As a general rule, however, a mild and soothing treatment is the most likely of all others to be beneficial; and any departure from it is only to be allowed in extreme cases, where the safety of the patient and the attendant justifies the application for a short period of the smallest possible amount of mechanical restraint.

Restraint must be always regarded as an evil, necessary at times, but never to be pushed beyond a certain limit. To make it the rule, and the opposite system the exception, is highly dangerous; and we hesitate not to affirm, that in most instances it would defeat its own object, if that were either the cure or the safe custody of the insane.

In our attempts to alleviate or to cure this dreadful disease, we must never forget that each patient presents us with an agent which we may use with wonderful effect, if we will avail ourselves of it, and that is, *the remains of the wreck*, which, to cease from metaphor, consists of some portion of the noble intellect, or of the moral sense, or of the instincts, which, if judiciously managed, may not only be itself repaired, but lead to the more perfect development of that which is obscured. In the treatment of the insane, the same management is required as that which is used with such marked results in the discipline of healthy minds; and no man can hope for success in this branch of medicine, who is not qualified to discern the character of the moral and intellectual powers, to gauge their strength, and then educate, by proper exercise, those which he discovers. To do this, there must be the best understanding between master and pupil. A marked solicitude for the welfare, the comfort, and the amusement of his patient, will gain his confidence, and enable the physician to bring into action that discipline by which he hopes to strengthen what remains of mental or moral power; from the improvement of which he further hopes to arrive at the complete mastery of the disease. But how can this plan of treatment be carried out, and all its capabilities tested, in an asylum where restraint, in the ordinary acceptation of the term, is practised and relied on as a remedial agent? So far from cherishing what remains, and awakening what is dormant, such a system, vigorously maintained, would but "quench the smoking flax and break the bruised reed." Happy is it, that what common sense, humanity, and Christianity teach us to do in the management of our fellow men, reduced by disease to the melancholy condition of the insane, both philosophy and practical experience have proved to be proper and right; and we think, if an inquiry into the practice of the world were made, it would be found that not *five per cent.* of those persons who are engaged in the management and cure of the insane, are in favour of an unqualified system of restraint. We do not, for one moment, suppose that Dr. Burnett would wish to be classed with this minority; and have no doubt that he is, in the main, favourable to the non-restraint principle; but it is unfortunate that he has laid himself open to the suspicion of his brethren by attacking the system of non-restraint, which he tells us is far from ultra, and which, because it is only partially adopted, he calls a deception. If, however, he holds the opinion, that a more vigorous and decided application of restraint, as a remedy for madness, is the best balm to sooth a wounded spirit, quiet a perturbed conscience, dissipate the incorporeal delusion, or awaken the faculties depressed or dormant, then we can only, in parting with this amiable and, we believe, excellent physician, express a hearty wish, that he may be successful in his arduous, but to our humble opinion, impracticable undertaking.

